# Birt-Hogg-Dube syndrome presenting as multiple oncocytic parotid tumors

**DOI:** 10.1186/1897-4287-10-13

**Published:** 2012-10-10

**Authors:** Noralane M Lindor, Jan Kasperbauer, Jean E Lewis, Mark Pittelkow

**Affiliations:** 1 Department of Health Science Research, Mayo Clinic Arizona; 2 Department of Otolaryngology; 3 Department of Laboratory Medicine and Pathology; 4 Department of Dermatology, Mayo Clinic Rochester

**Keywords:** Salivary gland, Oncocytic, Warthins tumor, Oncocytosis

## Abstract

Mutations in *FLCN* cause Birt-Hogg-Dubé syndrome, an autosomal dominant disorder notable for development of cutaneous fibrofolliculomas or trichodiscomas, a variety of renal tumors, and spontaneous pneumothorax due to cystic lung changes. We present a woman referred for genetic evaluation due to bilateral parotid gland tumors, who was subsequently diagnosed with Birt-Hogg-Dubé syndrome.

## Introduction

BHD syndrome is an uncommon genodermatosis characterized by the presence of multiple fibrofolliculomas, trichodiscomas, and acrochordons of the skin. There is significantly increased susceptibility to renal tumors, of which about half are hybrid chromophobe/oncocytic renal cancers; about 5% are oncocytomas. Pulmonary cysts occur in the majority of adults with BHD syndrome, leading to spontaneous pneumothorax in at least a quarter of affected individuals. A longer list of tumors have been reported rarely in BHD [1]; parotid tumors have been reported several times but have not served as the sentinel lesion bringing a patient to diagnosis.

### Case report

**Figure 1:**
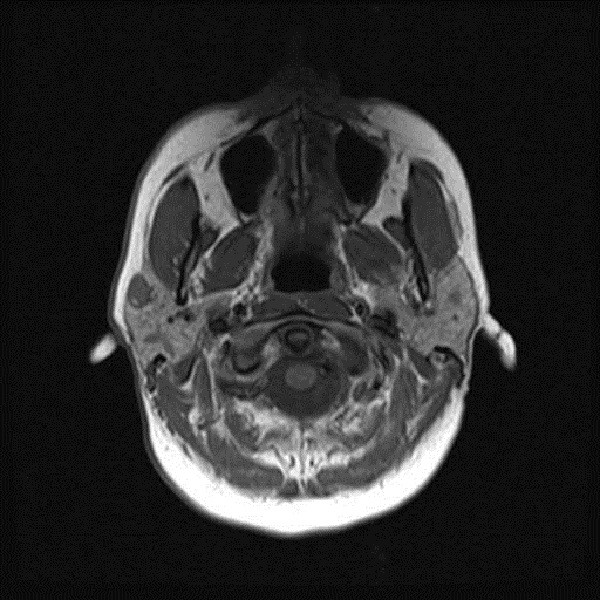
Axial T1 image demonstrating the largest right parotid nodule near the anterior edge of the gland with a small nodule in the posterior gland and a nodule in the left anterior parotid gland.

A previously healthy 45 year old Caucasian woman had a magnetic resonance scan for persistent mild hearing loss in her right ear. The cause for the hearing loss was not identified but the magnetic resonance imaging demonstrated multiple small parotid masses: a 9 mm diameter peripherally enhancing/T2 hyperintense lesion in anterolateral aspect of right parotid gland, a few additional smaller T1 hypointense nonenhancing lesions in right parotid gland and additional lesions in the superficial and deep lobes of the left parotid gland (Figure 1). Fine needle aspiration biopsies revealed a mildly hypercellular collection of epithelial cells with oncocytic differentiation and associated lymphoid aggregates; the interpretation was: suspicious for oncocytic neoplasm, favor Warthin tumor. The differential diagnosis included the spectrum of oncocytic proliferations which includes Warthin tumors, nodular oncocytic hyperplasia (nodular oncocytosis), and multiple oncocytomas of the parotid glands. The presence of lymphoid aggregates favors the diagnosis of Warthin tumors. The histologic diagnosis could not be further refined based upon the tissues obtained. Given the FNA findings and the distribution of the lesions, the plan is for observation of the parotid nodules until bothersome (size, cosmesis) and the treatment will require surgery addressing both the superficial and deep lobes of the parotid gland given the diffuse distribution of the nodules. She reported no prior history of tumors but had had several “moles” removed from her face for cosmetic reasons which were labeled only as “benign”. There was no history of spontaneous pneumothorax.

The family history was notable for maternal grandfather with prostate cancer, maternal grandmother with a bladder cancer diagnosed in her 40s and a lung cancer diagnosed in her 50s (she had smoked). A maternal uncle had a throat cancer and died at 58. The paternal family history was negative for neoplasms. Her three siblings and two children were apparently healthy.

**Figure 2:**
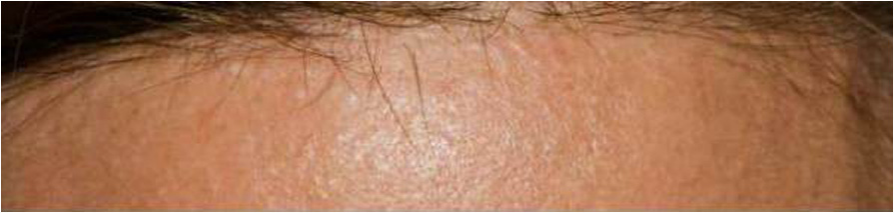
Inset: Whitish papules surrounding follicular orifice, especially prominent within anterior scalp hairline.

Her physical examination was normal except for a striking number of raised, smooth, flesh-colored cutaneous papules most notable around the scalp (Figure 2) and face in a generalized distribution and around the neck accompanied by numerous acrochordons. No intraoral lesions were noted. Skin biopsies showed findings of fibrofolliculomas in the scalp lesions and acrochordons on the neck. Imaging of the kidneys by computerized tomography showed only a single small cyst, but the bases of the lungs showed extensive cystic changes. Mutation analysis of *FLCN* revealed a c.779 + 1 G > T mutation which has been reported previously in BHD syndrome. Tissue from the biopsy was not available for further studies such as loss of heterozygosity of *FLCN*.

**Figure 3:**
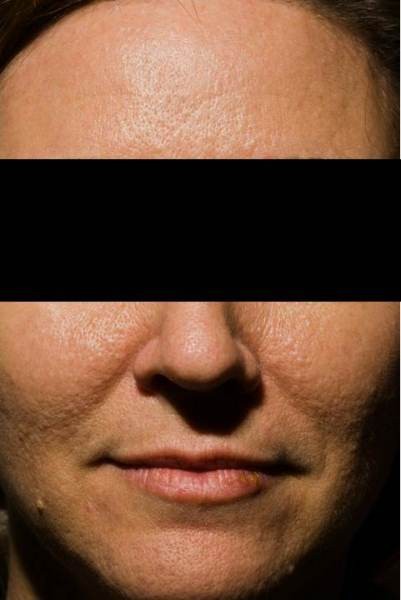
**Face and forehead.** Follicle-based, papular appearance of cheeks and forehead.

## Discussion

Parotid gland oncocytic lesions include a spectrum of histologic findings including nodular oncocytic hyperplasia, benign oncocytoma, and oncocytic carcinoma, and it may be reasonable to include Warthin tumor somewhere in this spectrum. Nodular oncocytic hyperplasia, if this is the correct description of the case comprises only 0.1% of parotid gland diseases [2] while 0.7% of 3,500 salivary gland tumors in another series contained benign oncocytic lesions [3]. There are a number of individual case reports suggesting that this disorder appears to be so uncommon as to warrant single case report presentations. Warthin tumors (also called papillary cystadenoma lymphomatosum, adenolymphomas, or cystadenolymphomas) are not so rare, being the second most common tumor of salivary glands (after pleomorphic adenomas), and characteristically affect older males, and are uncommonly bilateral.

A solitary parotid oncocytoma was reported in a 56 year old man with Birt-Hogg-Dubé's syndrome [4]. In 2005, Schmidt et al., reported on 219 individuals with BHD syndrome studied at that National Cancer Institute and parotid tumors were reported in two women (ages 62 and 72) and two men (ages 20 and 39). Three of these were oncocytomas but no information about multiplicity was provided [5]. Palmirotta et al., reported on two new BHD families and one woman had a parotid pleomorphic adenoma at age 43 [6]. In 2008, Toro et al., reported on 51 families (50 were new) with 89 gene mutation carriers and in this series two individuals has parotid gland oncocytomas,at ages 20 and 39 (it is not clear if this overlapped the Schmidt study or if the same ages were coincidental) [7]. Finally, Maffé et al., reported two parotid tumors among 19 patients with suspected Birt–Hogg–Dubé syndrome: a Warthin parotid tumor occurred in a 59 year old male in whom no *FLNC* mutation was found; and of most relavance to the current report, bilateral parotid tumors not otherwise specified, occurred at ages 32 and 43 years old in one man with a documented *FLNC* mutation [8].

Finding an oncocytic tumor in the parotid is of interest as the renal tumors which are characteristic of BHD often contain oncocytic cells. The relevance of this similarity is obscure. Oncocytic neoplasms, in general, are thought to result from cells that accumulate abundant mitochondria in their cytoplasm to form oncocytes via oncocytic metaplasia. They account for less than 1% of salivary gland tumors and can occur in other organs including kidneys, thyroid and parathyroid glands [9].

Molecular studies of Warthin tumors of the salivary glands indicate a recurrent t(11;19) translocation with associated *CRTC1-MAML2* fusion oncogene in a subset of Warthin tumors [10]. We were not able to study this biopsy for this finding. The chromosomal location of *FLCN* is 17p11.2 so no direct link to this translocated region is evident. Comparative genomic hybridization studies of salivary tumors did show some tumors with deletions involving 17p11.2 region in apparently 6 of 15 Warthin tumors but details in that publication were insufficient to determine if *FLCN* was deleted or not [11].

The function of *FLCN* is still a matter of investigation. Baba et al., [12] have studied the protein and stated that “the interaction of FLCN with FNIP1 and AMPK suggests a possible role for FLCN and FNIP1 in the nutrient/energy-sensing pathways involving AMPK and mTOR and may provide a molecular mechanism for the BHD phenotype”. No direct link to mitochondrial dysfunction has been proposed yet for this tumor suppressor gene but the oncocytic nature of the tumors in both kidney and now parotid gland raise this as a possibility.

Around half of BHD families recognized to date have a mutation in hot spot involving deletion (c.1285delC) or duplication (c.1285dupC) of a C nucleotide in the polycytosine tract in exon 11 of *FLCN*[5]. The mutation found in this patient is not in this area but has been reported previously in six individuals from two families [as IVS7 + 1] [7] and has not been reported since, per the Leiden Open Variome Database update of 2011 https://grenada.lumc.nl/LOVD2/shared1/variants.php?select_db=FLCN&action=view_unique. The major manifestation in these families generally appeared typical of BHD syndrome with renal tumors in five, lung cysts in six, documented fibrofolliculomas in five. Also reported was an angiofibroma, dermatofibrosarcoma protuberans, cutaneous leiomyosarcoma, and trichodiscoma. No parotid tumors were reported. Combined with the current case report, these families might be perceived as having more diverse dermatological findings than other BHD families, raising the possibility of some genotype-phenotype interaction.

Based upon the DNA diagnosis, the patient was counseled regarding autosomal dominant inheritance of this syndrome and the implications for multiple relatives, and was provided screening recommendations) for typical BHD syndrome with regard to renal and pulmonary complications, and she will remain under closer surveillance for changes in the parotid tumors and for additional dermatologic findings [13].

## Consent

Written consent for use of patient photographs were obtained.

## Competing interests

All authors’ have declared that they have no competing interest.

## Author contributions

All authors contributed to clinical diagnosis and review of the manuscript. All authors read and approved the final manuscript.
